# A comparison of scRNA-seq annotation methods based on experimentally labeled immune cell subtype dataset

**DOI:** 10.1093/bib/bbae392

**Published:** 2024-08-09

**Authors:** Qiqing Fu, Chenyu Dong, Yunhe Liu, Xiaoqiong Xia, Gang Liu, Fan Zhong, Lei Liu

**Affiliations:** Institutes of Biomedical Sciences, Fudan University, 200032 Shanghai, P.R. China; Institutes of Biomedical Sciences, Fudan University, 200032 Shanghai, P.R. China; Institutes of Biomedical Sciences, Fudan University, 200032 Shanghai, P.R. China; Institutes of Biomedical Sciences, Fudan University, 200032 Shanghai, P.R. China; Institutes of Biomedical Sciences, Fudan University, 200032 Shanghai, P.R. China; Intelligent Medicine Institute, Fudan University, 200032 Shanghai, P.R. China; Intelligent Medicine Institute, Fudan University, 200032 Shanghai, P.R. China

**Keywords:** scRNA-seq, cell-type annotation, benchmark

## Abstract

Cell-type annotation is a critical step in single-cell data analysis. With the development of numerous cell annotation methods, it is necessary to evaluate these methods to help researchers use them effectively. Reference datasets are essential for evaluation, but currently, the cell labels of reference datasets mainly come from computational methods, which may have computational biases and may not reflect the actual cell-type outcomes. This study first constructed an experimentally labeled immune cell-subtype single-cell dataset of the same batch and systematically evaluated 18 cell annotation methods. We assessed those methods under five scenarios, including intra-dataset validation, immune cell-subtype validation, unsupervised clustering, inter-dataset annotation, and unknown cell-type prediction. Accuracy and ARI were evaluation metrics. The results showed that SVM, scBERT, and scDeepSort were the best-performing supervised methods. Seurat was the best-performing unsupervised clustering method, but it couldn't fully fit the actual cell-type distribution. Our results indicated that experimentally labeled immune cell-subtype datasets revealed the deficiencies of unsupervised clustering methods and provided new dataset support for supervised methods.

## Introduction

Single-cell RNA sequencing is a significant technology helping to resolve biological questions at the single-cell level, greatly facilitating research in areas such as cancer and the immune system [1, 2]. Cell-type annotation is the foundation of scRNA-seq data analysis.

Cell-type annotation methods include unsupervised clustering and supervised annotation. Unsupervised clustering identifies similar cell groups based on gene expression by selecting features, reducing dimensions, and clustering. The cell groups are then annotated based on the differentially expressed genes of different clusters or the expression of marker genes. Supervised annotation involves training classification models on pre-labeled reference datasets and then annotating new datasets. With the development of numerous automatic single-cell annotation methods, methodological evaluation is beneficial for researchers to select appropriate methods based on their needs. There have been studies that evaluated single-cell annotation methods [3–6].

Those studies are based on single-cell datasets with known cell labels. However, the cell labels in commonly utilized single-cell datasets are predominantly derived through computational means, including methods like StemID [7], densityMclust [8], nearest neighbor graph clustering [9], hierarchical clustering [10], and Spearman correlation analysis [11]. These labels of cells are not from experimental measurements, and thus, it is possible that there are computational biases for the corresponding cell labels (Supplementary Table 4). Even in single-cell datasets with experimental cell labels, such as CellBench [12], which is composed of five distinctly different lung cancer cell lines, the complexity of the single-cell dataset is low. The majority of automatic cell annotation methods perform well on this CellBench [6]. However, in reality, single-cell datasets are much more complex, making it difficult to evaluate the real-world application of cell annotation methods. We believe that experimentally labeled single-cell immune cell-subtype datasets can avoid the computational biases of reference datasets caused by unsupervised clustering cell annotation methods. This approach can also more accurately reflect the effectiveness of methods in practical application. Furthermore, many deep learning-based methods have been developed, such as scBERT [13] and TOSICA [14], which still need further evaluation.

In response to the challenges above, compared with the CellBench dataset, this study introduced a more complex, experimentally labeled immune subtype single-cell dataset of the same batch for the first time. The dataset reflected the complexity of real-world conditions, aiming to evaluate current single-cell annotation methods. The study assessed 18 single-cell annotation methods, comprising 10 supervised and 8 unsupervised clustering approaches. Performance evaluations were conducted across five experimental scenarios: intra-dataset five-fold cross-validation, unsupervised clustering, immune cell-subtype validation, inter-dataset annotation, and prediction of unknown cell types. These evaluations were performed on four immune cell datasets. Accuracy was used as the evaluation metric for supervised annotations, while the Adjusted Rand Index (ARI) was employed for unsupervised clustering. Our study provided the specific guidelines for single-cell annotation methods based on experimentally labeled immune cell-subtype dataset.

## Methods

### Methods evaluation

This study screened and evaluated 10 supervised annotation methods and 8 unsupervised clustering methods. We selected the methods that performed well in the previous evaluation studies [3–6] and the latest deep learning-based methods. Those methods included SVM [6], SingleR [15], CHETAH [16], scmapcluster [17], scmapcell [17], as well as deep learning-based methods, such as ItClust [18], TOSICA [14], Cell BLAST [19], scBERT [13], scDeepSort [20], and unsupervised clustering methods Seurat (version 4.0.5) [21], monocle3 (version 1.0.0) [22], monocle2 (version 2.22.0) [23], SHARP [24], SC3 [25], scMAE [26], scFseCluster [27], scDeepCluster [28]. We summarized the normalization, feature selection, dimension transformation, algorithm, and rejection methods in detail (Supplementary Table 7).

### Datasets

We used our dataset—the Liu dataset and the other three datasets—the ZhengSort dataset, the Zheng68K dataset, and the PBMCbench dataset (Supplementary Table 3)—derived from peripheral blood immune cells. The composition of cell types is provided in Supplementary Table 5.

The Liu dataset was generated by our laboratory, where 150 mL of blood was drawn from a healthy individual who signed the informed consent of sample collection and data analysis. The study was approved by the Ethics Committee of the Institutes of Biomedical Sciences, Fudan University (No. 2018IBSJS014). Then, 10 immune cell types were obtained through positive and negative selection using antibody-coated magnetic beads from the isolated peripheral blood mononuclear cells (PBMCs). The dataset included CD4+ naive T, CD4+ memory T, CD8+ naive T, CD8+ memory T, NK, γδ T, naive B, memory B, granulocyte, and monocyte. These immune cells were then incubated with human pan-cell antibodies carrying specific nucleotide sequences (Supplementary Table 1) to label them. The labeled cells were pooled into one sample for sequencing, generating a single-cell sequencing dataset composed of mixed immune cell subtypes of the same batch. The information of immunomagnetic enrichment bead kits, immune cell surface proteins, and the theoretical purity of immune cells can be found in Supplementary Table 2.

The ZhengSort dataset was sampled from the different batches of reference datasets with the experimental labels, which were the single-cell sequencing datasets of 10 PBMC-sorted immune cells (CD14+ monocytes, CD19+ B cells, CD34+ cells, CD4+ helper T cells, CD4+/CD25+ regulatory T cells, CD4+/CD45RA+/ CD25− naive T cells, CD4+/CD45RO+ memory T cells, CD56+ natural killer cells, CD8+ cytotoxic T cells, CD8+/ CD45RA+ naive cytotoxic T cells) via magnetic bead enrichment. However, the reference dataset contained batch effects, and there was redundancy and overlap between the CD8+ Cytotoxic T-cell type and the CD8+/CD45RA+ Naive Cytotoxic cell type [11]. The ZhengSort dataset was available at https://zenodo.org/records/3357167.

The Zheng68K dataset was obtained from the same batch of single cell sequencing of peripheral mononuclear blood cells, whose labels were computational labels calculated through Spearman’s correlation with the same reference datasets as the ZhengSort dataset. Rather than using the cell labels provided by the authors, labels were re-annotated using the provided code (https://github.com/10XGenomics/single-cell-3prime-paper/tree/master/pbmc68k_analysis). The Zheng68K dataset originated from https://github.com/10XGenomics/single-cell-3prime-paper.

The PBMCbench dataset, whose labels were computational and obtained through the Louvain community detection algorithm and *AUC* calculation, included PBMC single-cell sequencing data obtained from low-throughput plate-based sequencing methods Smart-seq2, CEL-Seq2, and high-throughput sequencing methods 10x Chromium (v2), 10x Chromium (v3), Drop-seq, Seq-Well, and inDrops [29]. The PBMCbench dataset can be found at https://portals.broadinstitute.org/single_cell/study/SCP424/single-cell-comparisonpbmc-data.

### Dataset processing

In the analysis of the Liu dataset, given the less-than-perfect purity of magnetic bead purification, we adopted the purifying method utilized in the reference [11]. We initially normalized and standardized the raw data. Next, PCA dimension reduction was conducted on various cell types based on their experimental labels, with the selection of 30 principal components for subsequent *K*-means clustering into distinct clusters. After the initial cluster identification, refinement was achieved by discarding clusters based on the expression of specific marker genes, as illustrated in Supplementary Fig. 1. These included NK cells (*CD3E*-*CD4*-*NCAM1*+), granulocytes (*FCGR3B*+), monocytes (*CD14*+), memory CD4+ T cells (*CD3D* + *CD4* + *CCR7*−), naive *CD4*+ T cells (*CD3D* + *CD4* + *CCR7*+), memory *CD8*+ T cells (*CD3D* + *CD8B* + *CCR7*−), naive *CD8*+ T cells (*CD3D* + *CD8B* + *CCR7*+), γδ T cells (*CD4*-*CD8B*-*TRDC*+), memory B cells (*CD19 + CD79B + TNFRSF13B+*), and naive B cells (*CD19 + CD79B + TNFRSF13B*−). The analysis code named Liu_datasets_purified.R for the Liu data analysis was available at https://zenodo.org/records/10947879. This process culminated in the generation of a refined reference dataset called the Liu dataset (Supplementary Fig. 3).

In the ZhengSort dataset and the Zheng68K dataset analysis, genes not expressed across all cells were filtered out. We calculated the median and the median absolute deviation (MAD) of the total gene expression of cells, then removed cells whose total gene expression fell below Median − 3 × MAD.

In the PBMCbench dataset analysis, we selected the entry with the highest gene expression value for duplicate gene symbols. Given that this dataset had already been pre-annotated following the exclusion of low-quality cells and the inherently small number of cells in low-throughput sequencing datasets, no additional cells were removed.

### Experimental design

This study aimed to evaluate the efficacy of annotation methods within actual datasets, thus excluding the use of simulated datasets. The evaluation of annotation methodologies encompassed intra-dataset analysis, immune subtypes annotation, unsupervised clustering, inter-dataset annotation, and the prediction of known and unknown cell types.

### Intra-dataset validation

For the supervised methods, we evaluated the performance by applying the five-fold cross-validation across the processed data——Liu, Zhensort, and Zheng68K dataset. The dataset was divided into five-folds in a stratified manner so that each cell population in each fold was equal. The four-fold and the remaining one-fold data were used as the training and test datasets. During validation, because of the minimal number of CD4+ helper T cells in Zheng68K, scBERT training failed. So, we removed the CD4+ helper T cells of Zheng68K only for scBERT and compared this result with other methods.

### Immune subtype validation

We extracted immune subtypes from each dataset, where we deleted the non-T-cell subtypes of the Liu dataset and non-CD4+ T cells of the Zheng68K dataset. The absolute numbers of T cells of the Liu dataset and CD4+ T cells of the Zheng68K dataset were not altered, but the relative proportion of each T-cell subtype was elevated. The remaining dataset was divided into five-folds in a stratified manner so that each cell population in each fold was equal. The four-fold and the remaining one-fold data were used as the training and test datasets, respectively.

### Inter-dataset prediction

We adopted the PBMCbench dataset for the inter-dataset prediction, including seven sequencing protocols. In the first scenario, we trained on the pbmc1 samples with the other six protocols and tested on the other protocol. In the second scenario, we trained on the pbmc1 sample and tested on the pbmc2 sample using the same protocol.

### Unsupervised clustering

We constructed the computational reference dataset Zheng68K_sampled dataset using sample() for random downsampling from the Zheng68K dataset, with cell-type proportions identical to Zheng68K, excluding a very small number of CD4+ helper T cells. Then, we evaluated the performance of unsupervised clustering in the Liu dataset and Zheng68K_sampled dataset. For methods based on R, we used FindClusters() for Seurat, cluster_cells() for monocle3, clusterCells() for monocle2, sc3() for SC3, and SHARP() for SHARP. For methods based on Python, we obeyed the tutorial of a deep learning model to get the results. Because the cell-type number was known, we generated a 10-cluster result for the Liu and Zheng68K_sampled dataset. However, this kind of result did not perform well in the subtype distinction and metrics evaluation. So, we generated 11- and 12-cluster results and finally chose the 11-cluster result for subtype distinction comparison.

### The prediction of known and unknown cell types

We stratified the Liu dataset into five-folds. Then, four-folds were treated as the training dataset and the other one-fold as the testing dataset. We removed the T cells, CD4+ T cells, and naive CD4+ T cells from the training dataset in different experiments and kept the testing dataset covering all cells. The three experiments were conducted on all the supervised methods with rejection options.

### Evaluation metrics

The main metric for supervised cell annotation performance is accuracy. That for unsupervised clustering methods is *ARI*.

## Results

### scRNA-seq dataset of experimentally labeled immune cell subtypes of the same batch

**Figure 1:**
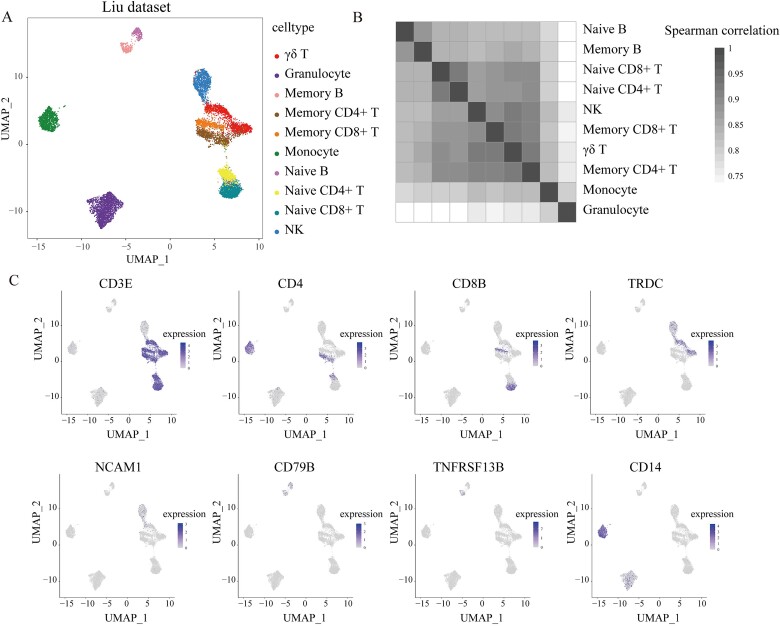
The composition of the Liu dataset (A) UMAP distribution of experimental labels for cell types in the Liu dataset. (B) Spearman correlation of gene expression for different immune cell subtypes in the Liu dataset. (C) Distribution of marker gene expression by cell type.

Firstly, we constructed an evaluation dataset of immune cell subtypes with experimental labels of the same batch through mixed single-cell sequencing, including CD4+ naive T, CD4+ memory T, CD8+ naive T, CD8+ memory T, NK, γδ T, naive B, memory B, granulocyte, and monocyte (Methods). We determined the distribution of cell types through classic cell markers. The distribution of experimentally labeled cell types was consistent with the marker gene expression. The same experimental labels almost clustered together, indicating the reliability of the data (Fig. 1C). Consistent with literature [30–32], our dataset also showed NK cells expressing *TRDC*, monocytes expressing *CD4*, and neutrophils expressing *CD14*, further attesting to its reliability. We found that the clustering results for monocytes, granulocytes, B cells, NK, and T cells were distinctly separated. In contrast, within B-cell subgroups (naive B, memory B) and T-cell subgroups (naive CD4+ T, naive CD8+ T, memory CD4+ T, memory CD8+ T, γδ T), boundaries exhibited intermixing among cells, and Spearman correlation analysis showed high similarity within B cell subgroups and T-cell subgroups (Spearman correlation >0.8), making it difficult to distinctly differentiate between cell subgroups (Fig. 1B).

### Intra-dataset validation

**Figure 2:**
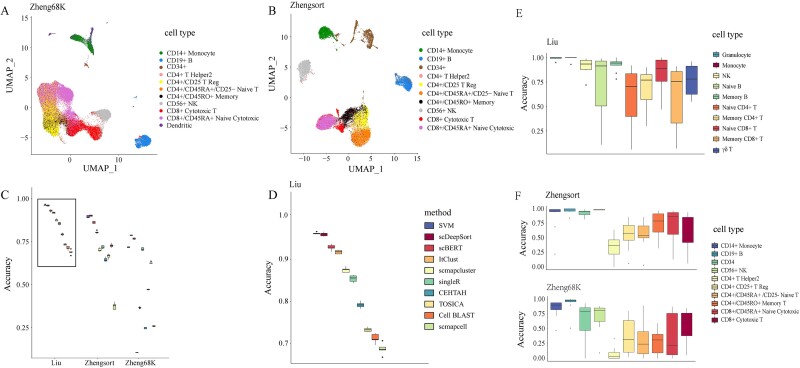
Intra-dataset validation. (A) Cell-type distribution in the Zheng68K dataset. (B) Cell-type distribution in the ZhengSort dataset. (C) Accuracy of cell-type annotation methods across three datasets. (D) The enlarged view of the blackbordered part (the Liu dataset) in C. (E–F) Annotation accuracy for each cell type across the three datasets.

To assess the impact of experimental datasets on the performance evaluation of annotation methods, we compared the Liu dataset with other commonly used peripheral blood immune cell datasets, Zheng68K and ZhengSort dataset (Fig. 2A, B).

The Zheng68K dataset comprised 11 immune cell types, while the ZhengSort dataset contained identical cell types but with a better data balance. Analysis revealed that 10 supervised cell annotation methods achieved higher accuracy on the Liu dataset than the Zheng68K dataset, with accuracy rates surpassing 0.6. Apart from Cell BLAST, these methods also showed superior accuracy on the Liu dataset over the ZhengSort dataset (Fig. 2C, Supplementary Fig. 5). Also, the performance on the Liu dataset performed better than the Zheng68K dataset. Compared with the Zheng68K dataset, the ZhengSort and Liu datasets shared a commonality regarding the approximately equal distribution of sample numbers across categories within the datasets (Supplementary Fig. 4). Research indicated that the efficiency of supervised annotation methods improves with better data balance in the training set [5]. Among the three datasets, SVM, scBERT, and scDeepSort emerged as the most effective single-cell annotation methods. They consistently ranked among the top three in annotation accuracy across all datasets, achieving accuracies as high as 0.95 (Fig. 2D, Supplementary Table 6, Supplementary Figs 6 and 8).

Despite employing supervised methods, the accuracy for annotating T-cell subtypes, including CD4+ T cells, remained notably low (Fig. 2E–F, Supplementary Table 6). Given the challenge of altering the absolute number of cells within datasets, we strategically removed non-T-cell subtypes from the training set. This approach aimed to increase the relative proportion of each T-cell subtype, thereby adjusting the data balance within the reference dataset to assess its impact on T-cell subtype annotation accuracy. Specifically, within the Liu dataset, T-cell subtypes were isolated for annotation, revealing that methods such as SVM, scBERT, and scDeepSort did not exhibit significant changes in accuracy for T-cell subtypes. Conversely, Cell BLAST and ItClust showed improved accuracy. Similarly, applying this approach to the Zheng68K dataset led to slightly increased accuracy for SVM, scBERT, Cell BLAST, and ItClust (Supplementary Fig. 10). These findings suggested that for cell subtypes with poor data balance in training datasets, isolating and specifically annotating those cell types only improved accuracy slightly.

### Unsupervised clustering

**Figure 3:**
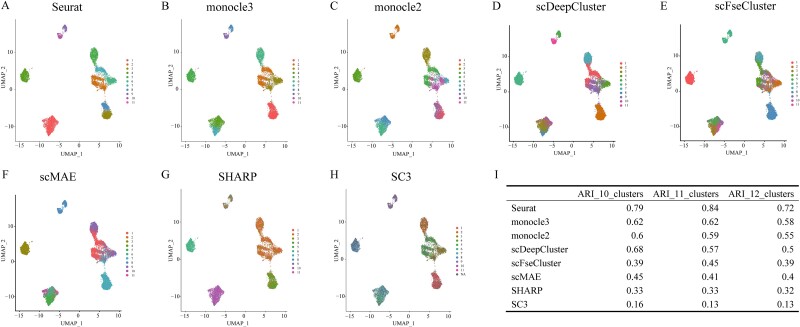
Performance of unsupervised clustering methods. (A–H) Performance of unsupervised clustering methods on the Liu dataset. (I) ARI values for different numbers of clusters, where ARI_10_clusters represent the ARI value for unsupervised clustering into *n* clusters (*n* = 10, 11,12), ARI_n_clusters represent the ARI value for unsupervised clustering into *n* clusters.

Accurate single-cell annotation can significantly reduce scRNA-seq analysis workload. However, current methods still involve annotating cell types through marker genes or differential genes after unsupervised clustering. There is still a lack of comparison for unsupervised clustering methods using datasets of immune cell subgroups with experimental labels. Therefore, to evaluate the performance of unsupervised single-cell clustering methods on experimentally labeled datasets, we assessed eight state-of-the-art methods [5] using the Liu dataset. The results showed that the best-performing unsupervised method was Seurat, followed by monocle3 and monocle2, while SC3 and SHARP performed poorly (Fig. 3I, Supplementary Fig. 11). The UMAP visualization results revealed that Seurat can distinguish the immune cell subtypes, including naive B, memory B, naive CD4+ T, and memory CD4+ T. Monocle3 and scDeepCluster can distinguish naive B and memory B, while the other unsupervised clustering methods failed (Fig. 3). However, Seurat did not classify all γδ T subtypes into the same category and distinguish between the memory CD4+ T and memory CD8+ T-cell subgroups (Fig. 1A). Therefore, none of these five unsupervised clustering methods can perfectly distinguish these three cell subgroups (Fig. 3A–H). We also evaluated the methods’ performance in the computational Zheng68K_sampled dataset, and the boundary of subtypes was obviously blurred. We found that all the methods showed worse results (ARI < 0.5) compared with the Liu dataset (Methods and Supplementary Fig. 12).

### Inter-dataset annotation

**Figure 4:**
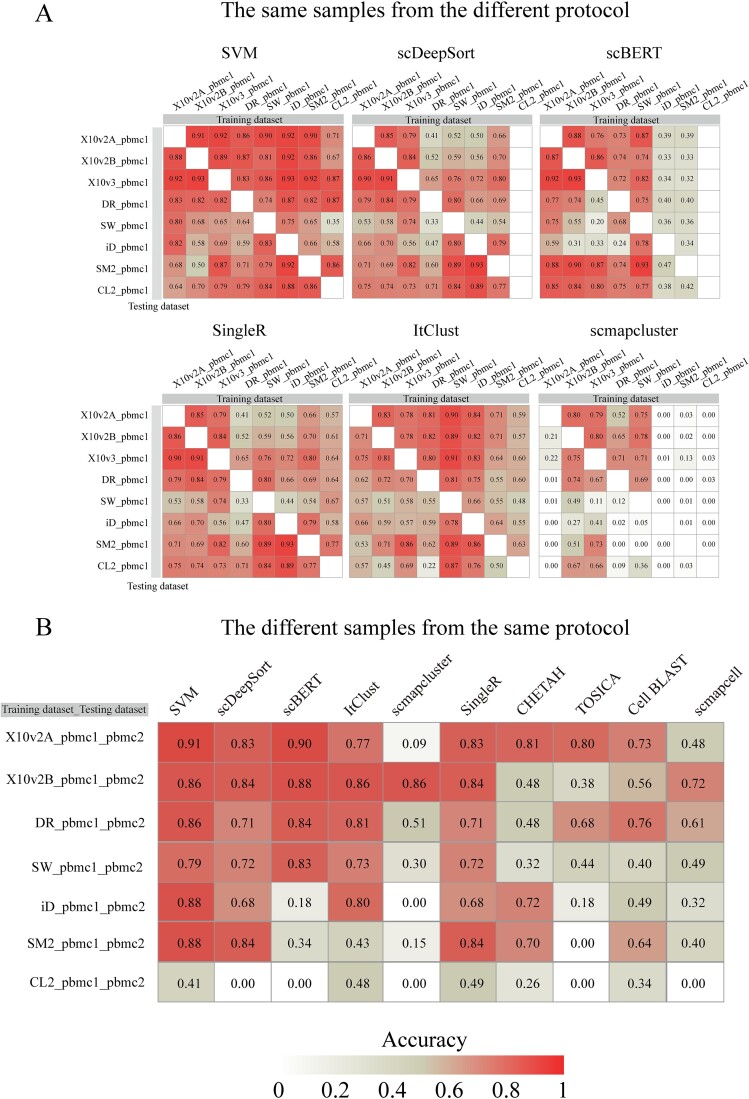
Inter-dataset validation. (A) Accuracy of methods for the same sample under different sequencing protocols, with columns representing training sets, rows representing test sets, and values indicating the accuracy for corresponding training and test sets. Blanks indicate failure of training. SM2 stands for Smart-seq2, X10v2A for 10x Chromium(v2)_A, 10xChromium(v2)_B for X10v2_B, CL2 for CEL-Seq2, DR for Drop-Seq, iD for inDrops, and SW for Seq-Well. (B) Accuracy of methods for the same tissue under the same protocol across different samples, SW_pbmc1_pbmc2 indicates using SW_pbmc1 as the training set and SW_pbmc2 as the test set.

A common application scenario is inter-dataset annotation, and currently, there is still a lack of experimentally labeled multi-batch single-cell datasets. To assess the performance of supervised methods in inter-dataset annotation, we used the PBMCbench immune cell dataset (Supplementary Fig. 13). Inter-dataset annotation included two scenarios: one was the same sample with different sequencing schemes, and the other was different samples with the same sequencing scheme. In the first scenario, we found higher accuracy for specific pairs of protocols. For instance, methods performed well when trained on 10X2A and then tested on 10Xv2B and 10Xv3, and vice versa. Remarkably, most methods had higher accuracy when trained on iD and tested on SM2 (accuracy > 0.85). We also found that the choice of training dataset was critical, and the methods trained on SM2 and iD generally resulted in lower accuracy. Given the predominance of 10X sequencing in single-cell methods, practical considerations suggested SVM, scBERT, scDeepSort, and SingleR as the most effective for inter-dataset annotation (Fig. 4A). In the second scenario, we found that the overall high-accuracy methods were SVM, followed by scBERT, then ItClust, and scDeepSort. Most methods performed well when trained and tested on 10Xv2 protocol, followed by DR, SW, iD, SM2, and CL2, which indicated that the choice of sequencing protocols was essential (Fig. 4B, Supplementary Fig. 14). Combining the results of two cross-dataset annotation scenarios, we suggested that the methods with high annotation accuracy were SVM, scBERT, and scDeepSort.

### Predicting known and unknown cell types

**Figure 5:**
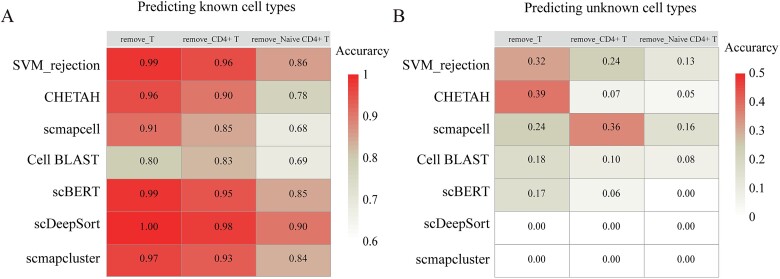
Accuracy of predicting known and unknown cell types. (A) Accuracy of predicting known cell types. (B) Accuracy of predicting unknown cell types, remove_T for removing T cells, remove_CD4+ T for removing CD4+ T cells, remove_Naive CD4+ T for removing Naive CD4+ T in the training dataset and keeping intact in the testing dataset.

The training set may not always include all cell types existing in the test set. Thus, accurately predicting known and unknown cell types enabled researchers to discover new cell subgroups. To assess the effectiveness of methods in such a task, we evaluated these methods using the Liu dataset. Methods capable of predicting unknown cell types included SVM_rejection, CHETAH, scmapcell, scmapcluster, Cell BLAST, scBERT, and scDeepSort. Given that the Liu dataset contains T-cell subgroups, we conducted tests in three scenarios: (1) the training set excluded T cells, (2) the training set excluded CD4+ T cells; and (3) the training set excluded naive CD4+ T cells. The test set included all cell types. Ideally, methods would accurately annotate positive samples while correctly excluding negative samples. In practice, with the gradual removal of T cells, CD4+ T cells, and naive CD4+ T cells, the accuracy of annotating known cell types gradually decreased and showed a significant decrease after removing naive CD4+ T cells because learning the fine features for distinguishing removed and unremoved cells became difficult. SVM, scDeepSort, scBERT still had the highest annotation accuracy (Fig. 5A). Also, the accuracy of predicting unknown cells gradually decreased; with the removal of T cells, CHETAH and SVM_rejection had the highest accuracy in annotating unknown cell types, but the accuracy was <0.5 (Fig. 5B). In summary, as the difficulty of cell annotation increased, the accuracy for both known and unknown cell types gradually decreased. Above all, the best-performing methods were SVM_rejection and CHETAH. These results indicated that accurately annotating both known and unknown cell types with single-cell annotation methods remained challenging.

## Discussions and conclusions

This study selected 18 single-cell annotation methods, including 10 supervised and 8 unsupervised methods. The results suggested using SVM, scBERT, and scDeepSort for supervised single-cell annotation and Seurat for unsupervised clustering. This study provided specific guidelines for single-cell annotation methods.

Intra-dataset validation and inter-dataset annotation revealed that SVM, scBERT, and scDeepSort exhibited the highest accuracy. Abdelaal T’s studies demonstrated SVM’s strong performance, aligning with our results [6]. SVM is noted for robust generalization capabilities, effective learning from limited training samples and accurate prediction of testing samples. This study also underscores the impressive performance of deep learning methods like scBERT and scDeepSort [20]. These models remain challenging to interpret fully. scDeepSort leveraged weighted graph neural networks, and GNNExplainer revealed that scDeepSort could identify significant subgraph structures and node features corresponding to highly expressed genes and marker genes in specific cell types, which facilitated the nuanced characterization of cell types through the cell-gene graph iteration. scBERT employed multi-head attention mechanisms and self-supervised learning, initially processing vast amounts of unlabeled single-cell data to discern gene expression patterns and interactions, enhancing model performance and interpretability. The efficacy of these deep learning approaches likely stems from their ability to elucidate gene relationships, thereby capturing cell-type characteristics accurately.

In supervised annotation, the obtained conclusions were consistent between datasets with and without experimental labels. This conclusion indicated that the discrepancy between computational and experimental reference datasets did not affect the performance evaluation of supervised methods, which aligned with previous speculations that the random errors of computational labels should not have systematic impact on the evaluation [33]. SVM, scBERT, and scDeepSort performed well on annotating T-cell immune subtypes. We found that the cell annotation method TOSICA [14], which was trained by the knowledge-based mask matrix from Gene Set Enrichment Analysis (http://www.gsea-msigdb.org/gsea/downloads.jsp), was difficult to distinguish between subtypes of cells with similar functions, which might indicate that biological functional gene sets for classification did not performed well.

In unsupervised clustering, we observed that γδ T cells, memory CD4+ T cells, and memory CD8+ T cells could not be clustered accurately, the distribution of which was not conformed to a dense and circular pattern around the centroid, as seen in other cell types. Instead, their distribution leaned towards a rectangular shape. This discrepancy suggests that existing single-cell unsupervised clustering methods, which yield circular clusters, may struggle with non-circular distributions. Addressing this limitation represents a potential area for unsupervised clustering methods.

However, in the annotation of unknown cell types, Abdelaal [6] discovered that in the Zheng68K dataset, the removal of similar T-cell subtypes (such as CD4+ and memory T cells) for accuracy of annotating unknown cell types did not show a decline. Conversely, the accuracy declined in the Liu dataset, as expected. Moreover, we found that the Zheng68K dataset might have errors in differentiating CD4+ T-cell subtypes, especially naive CD4+ T cells, because the labels were dependent on the Spearman correlation with a reference dataset, which contained errors of redundancy in cell types, such as CD8+ cytotoxic T and CD8+/CD45RA+ naive cytotoxic T. Also, This method risked incorrect labeling because more than 11 immune cell types defined by Zheng68K exist in the peripheral blood, suggesting potential inaccuracies in classifying both known and unknown cells. Additionally, Abdelaal [12]‘s research design, which involved excluding specific immune cells from the training set and then only testing on those excluded cells, cannot accurately assess the method’s effectiveness in annotating cells that remained in the training set. Our study, in contrast, includes the removed and non-removed cell types. The findings reveal a contradiction in the precision of predicting known and unknown cell types, presenting a challenge in single-cell annotation.

However, this study still has limitations. Initially, the magnetically pre-enriched cells with less than 100% purity for constructing experimentally labeled datasets complicate data processing. Flow cytometry, offering higher purity cell sorting, represents a preferable alternative. However, it is worth noting that high-pressure cell sorting of flow cytometry may cause some cell damage, which may lead to more cell rupture. Additionally, it comes with a high cost, demands skilled personnel, and involves complex sample fluorescent labeling. [34, 35]. In contrast, immunomagnetic bead purification has lower purity but preserves cell viability. Meanwhile, it is less expensive, more user-friendly, and simplifies protocols [36, 37]. Therefore, the selection of purification methods depends on practical conditions. Additionally, cell rupture introduces RNA from lysed cells into the solution, complicating initial data processing [38–40]. Analyzing T-cell subtypes is challenging as CD45RA+ and CD45RO+ are both derived from the *PTPRC* gene, only based on which it is still difficult to distinguish naive from memory T cells. Instead, *CCR7* is used as the marker gene, differentiating naive T (*CCR7*+) from memory T (*CCR7*−), albeit reducing memory T-cell amounts due to the inclusion of central memory T cells (CCR7 + CD45RA−) [41–43]. In analyzing B cell subtypes, flow cytometry differentiates naive B (CD27−) from memory B (CD27+) cells by CD27 expression [44]. However, this study reveals that memory B cells exhibit low *CD27* expression (Supplementary Fig. 2), blurring the distinction from naive B cells, possibly due to transcriptional under-expression or sequencing capture inefficiency [45]. Consequently, *TNFRSF13B* highly expressed in memory B cells, was used for distinction [46]. For inter-dataset cell annotation, the lack of experimentally labeled immune cell subtypes of multiple batches led to the work on inter-dataset single-cell annotation being incomplete, only using the PBMCbench dataset.

In summary, this study indicated that in supervised methods, the impact of datasets labeled either experimentally or computationally on the outcomes was minimal. However, for unsupervised clustering, datasets with experimental labels more accurately reflected the clustering results. We recommend using SVM, scBERT, and scDeepSort as choices for supervised annotation methods, and Seurat for unsupervised clustering methods.

**Key Points**

- We first constructed an experimentally labeled immune cell-subtype single-cell dataset of the same batch, which was better than the previous reference dataset.
- Our study indicated that the discrepancy between computational and experimental reference datasets did not affect the performance evaluation of supervised methods.
- We systematically evaluated 18 cell annotation methods and showed that SVM, scBERT, and scDeepSort were the best-performing supervised methods. Seurat was the best-performing unsupervised clustering method, but it couldn't fully fit the actual cell-type distribution.

## Supplementary Material

Supplementary_Figure_1_bbae392

Supplementary_Figure_2_bbae392

Supplementary_Figure_3_bbae392

Supplementary_Figure_4_bbae392

Supplementary_Figure_5_bbae392

Supplementary_Figure_6_bbae392

Supplementary_Figure_7_bbae392

Supplementary_Figure_8_bbae392

Supplementary_Figure_9_bbae392

Supplementary_Figure_10_bbae392

Supplementary_Figure_11_bbae392

Supplementary_Figure_12_bbae392

Supplementary_Figure_13_bbae392

Supplementary_Figure_14_bbae392

Supplementary_Table_bbae392

Supplementary_Table_6_bbae392

Supplementary_Table_7_Summary_of_methods_bbae392

## Data Availability

The Liu dataset contains 10 types of purified immune cells, and the original dataset can be accessed via PRJNA843175, available as of the publication of this article. The processed data can be downloaded from Zenodo (https://zenodo.org/records/10947879) and is available as of the publication of this article. The code for this study is available at https://github.com/Qiqing-Fu/single-cell-annotation-methods.
