# Supplementary figures and images for: A comparison of scRNA-seq annotation methods based on experimentally labeled immune cell subtype dataset
Source: Brief Bioinform. 2024 Aug 9;25(5):bbae392. doi: 10.1093/bib/bbae392 (PMC11312369; doi:10.1093/bib/bbae392)

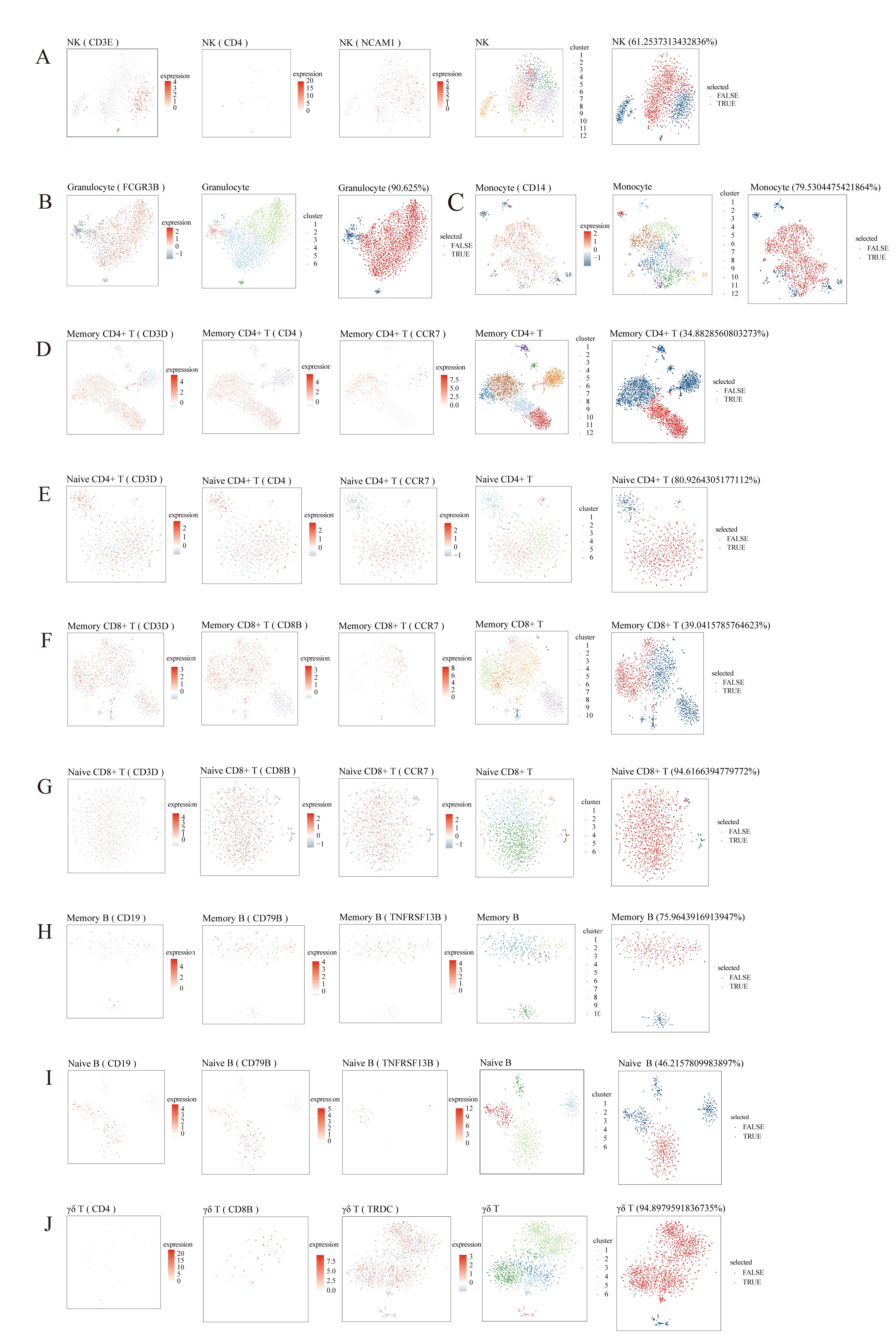

Supplement: Supplementary_Figure_1_bbae392 [file supplementary_figure_1_bbae392.jpeg]

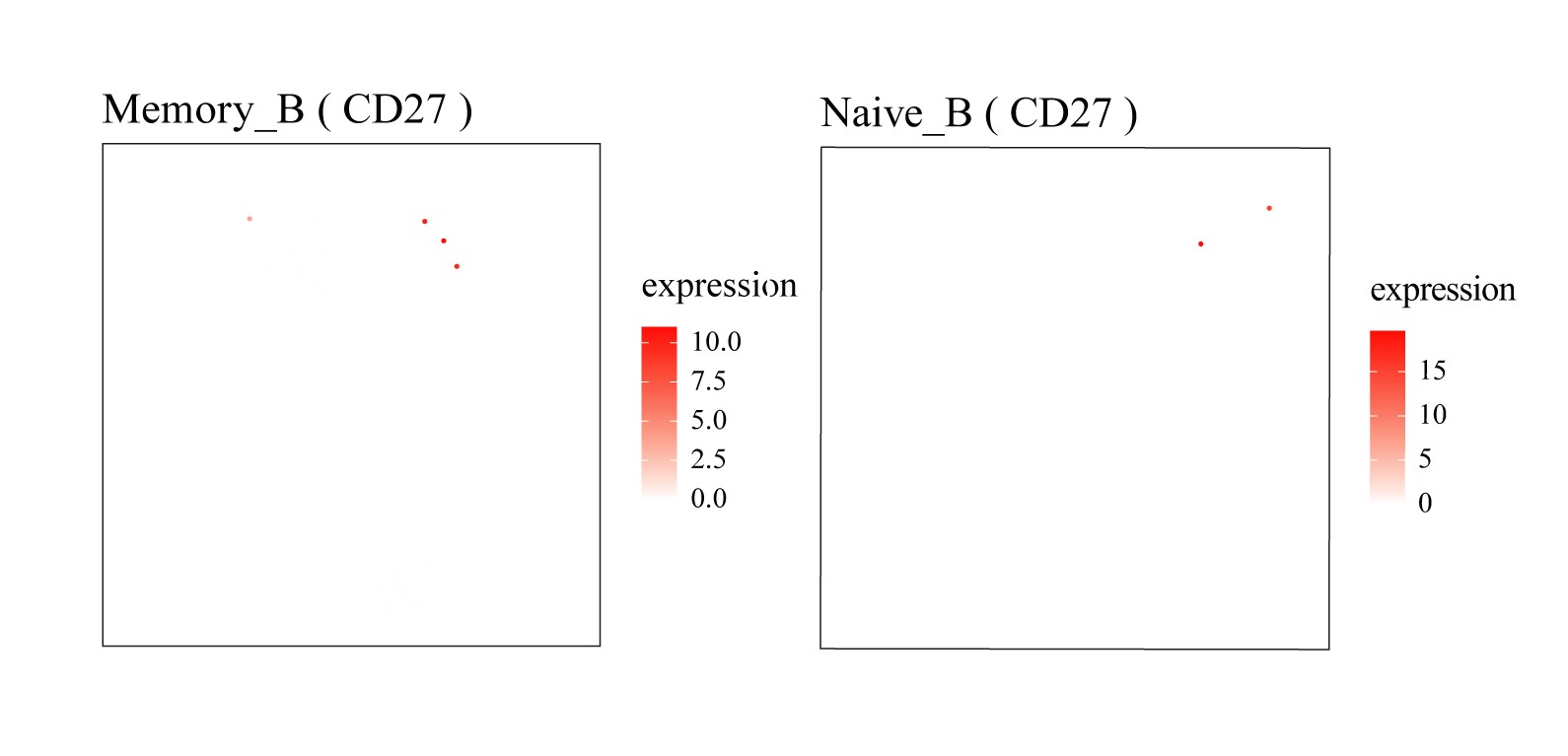

Supplement: Supplementary_Figure_2_bbae392 [file supplementary_figure_2_bbae392.jpeg]

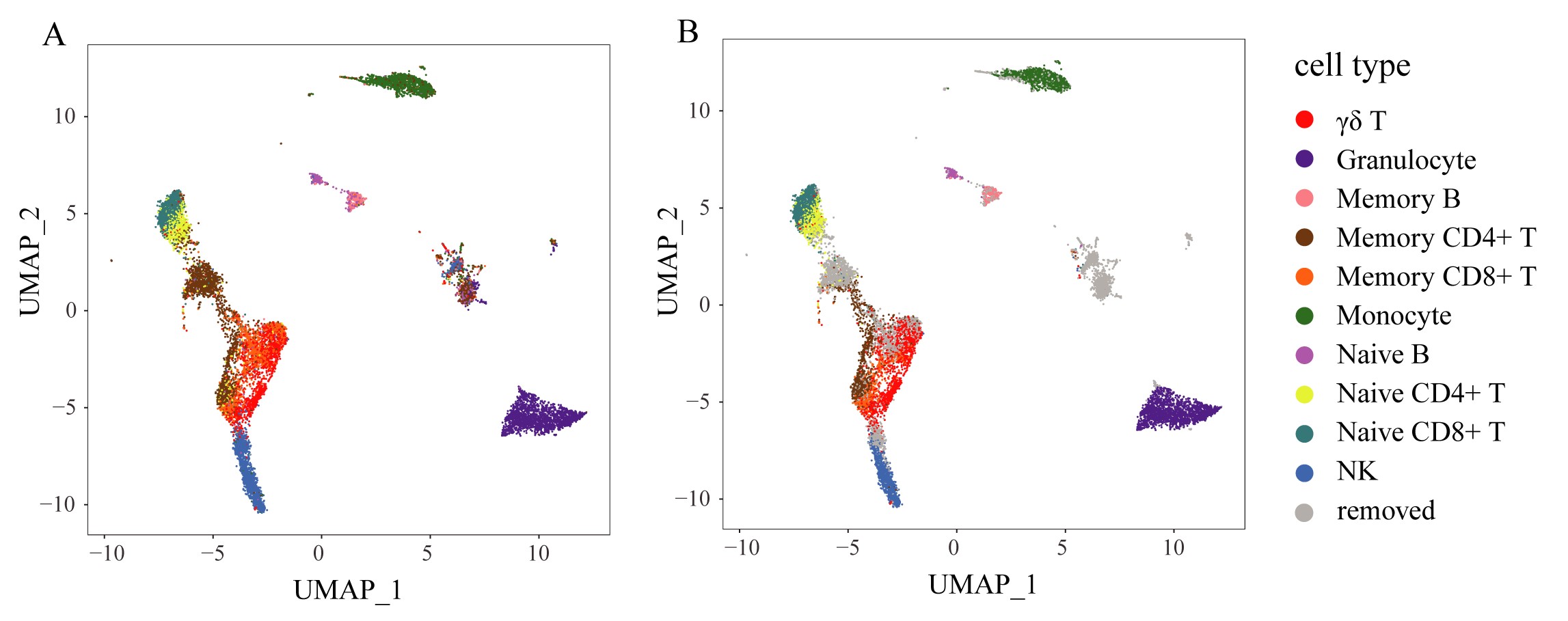

Supplement: Supplementary_Figure_3_bbae392 [file supplementary_figure_3_bbae392.jpeg]

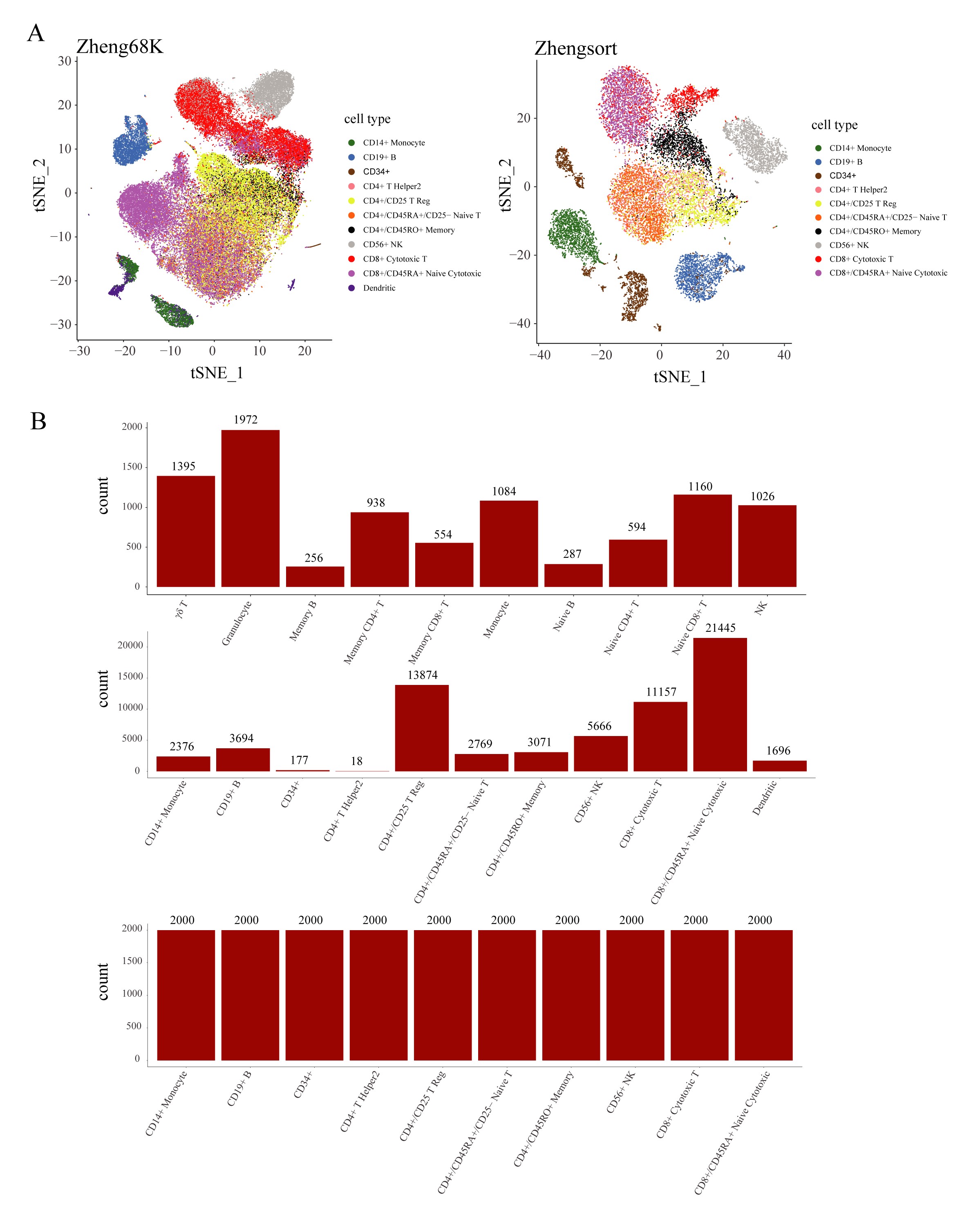

Supplement: Supplementary_Figure_4_bbae392 [file supplementary_figure_4_bbae392.jpeg]

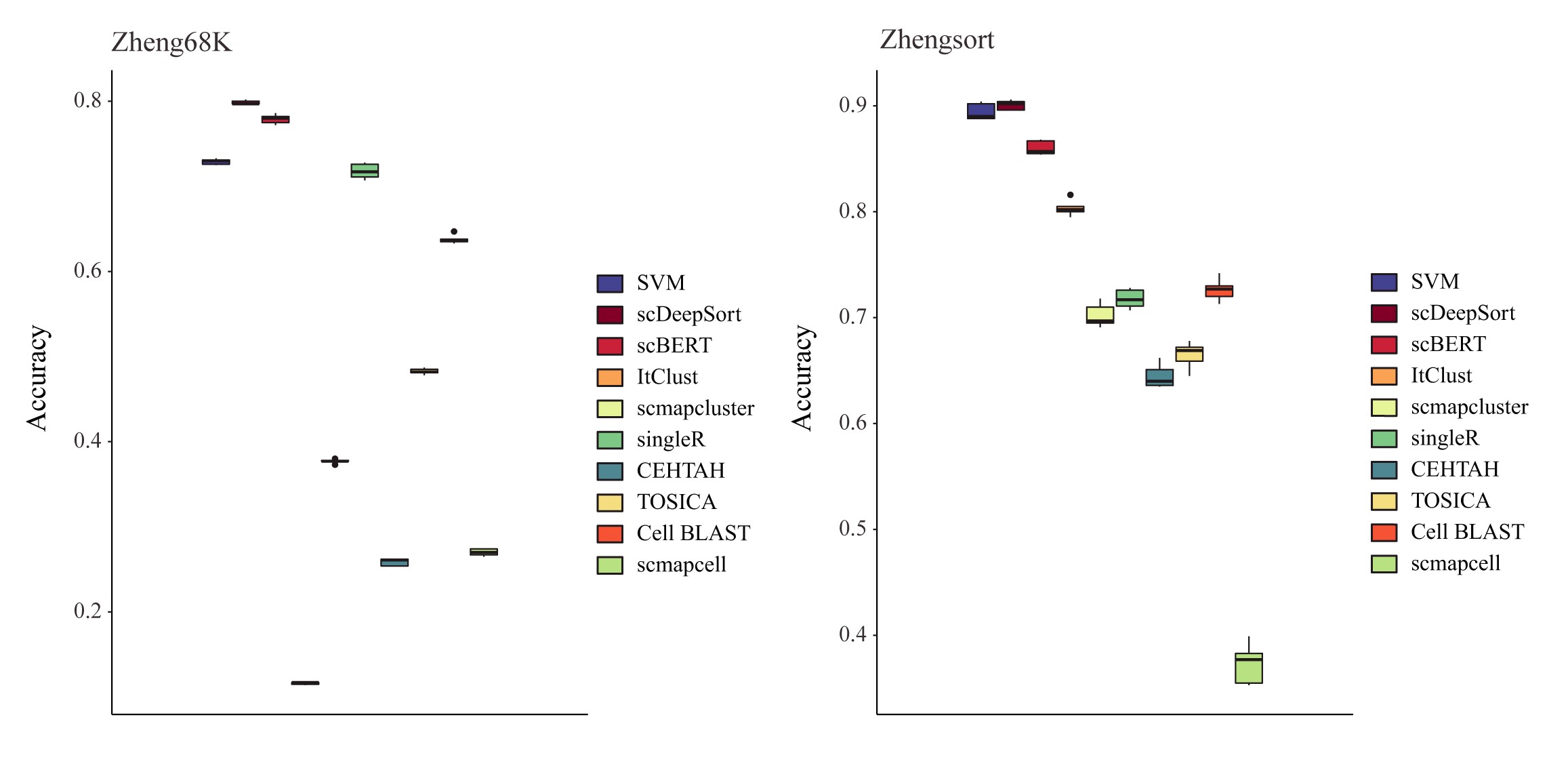

Supplement: Supplementary_Figure_5_bbae392 [file supplementary_figure_5_bbae392.jpeg]

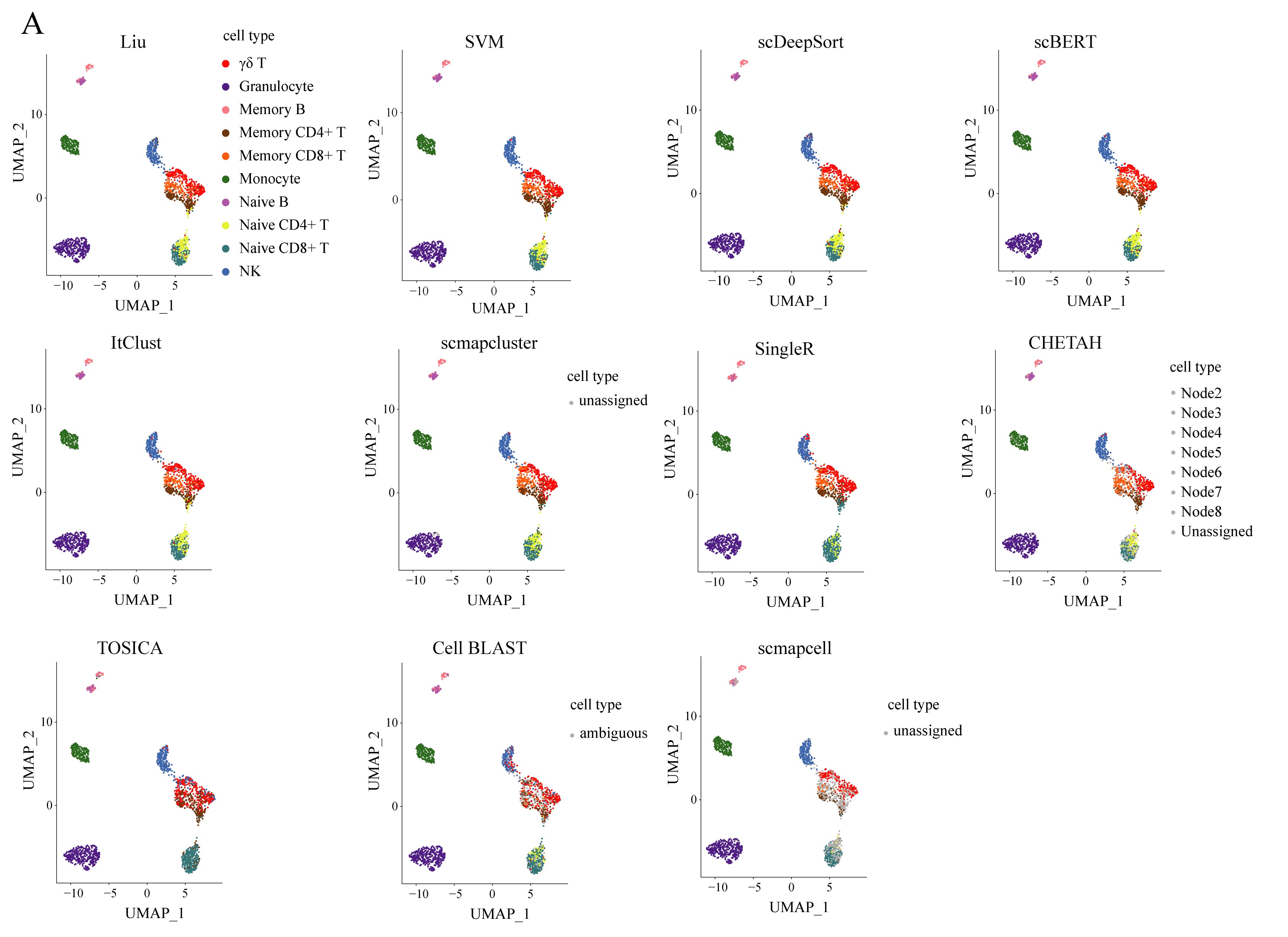

Supplement: Supplementary_Figure_6_bbae392 [file supplementary_figure_6_bbae392.jpeg]

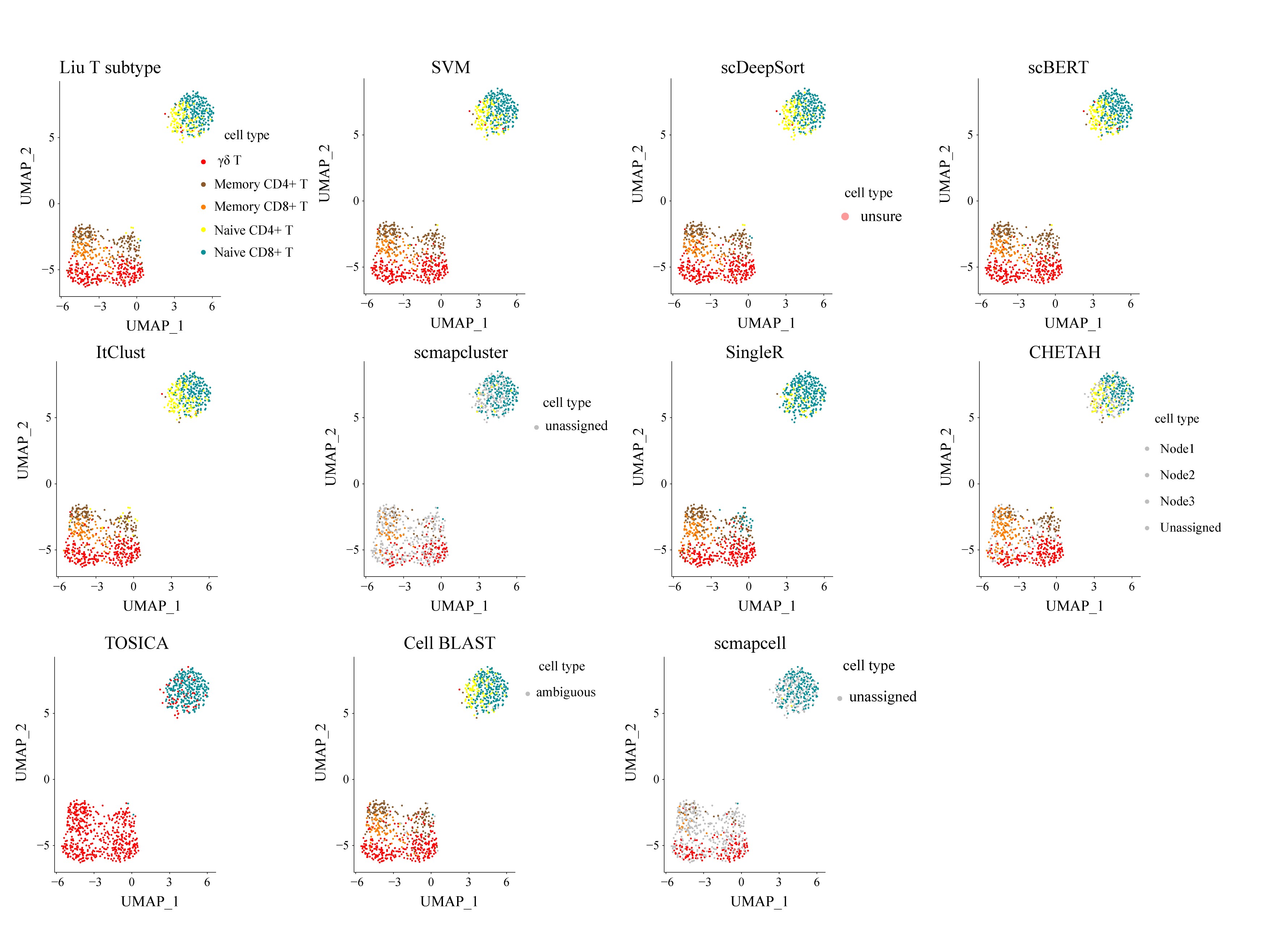

Supplement: Supplementary_Figure_7_bbae392 [file supplementary_figure_7_bbae392.jpeg]

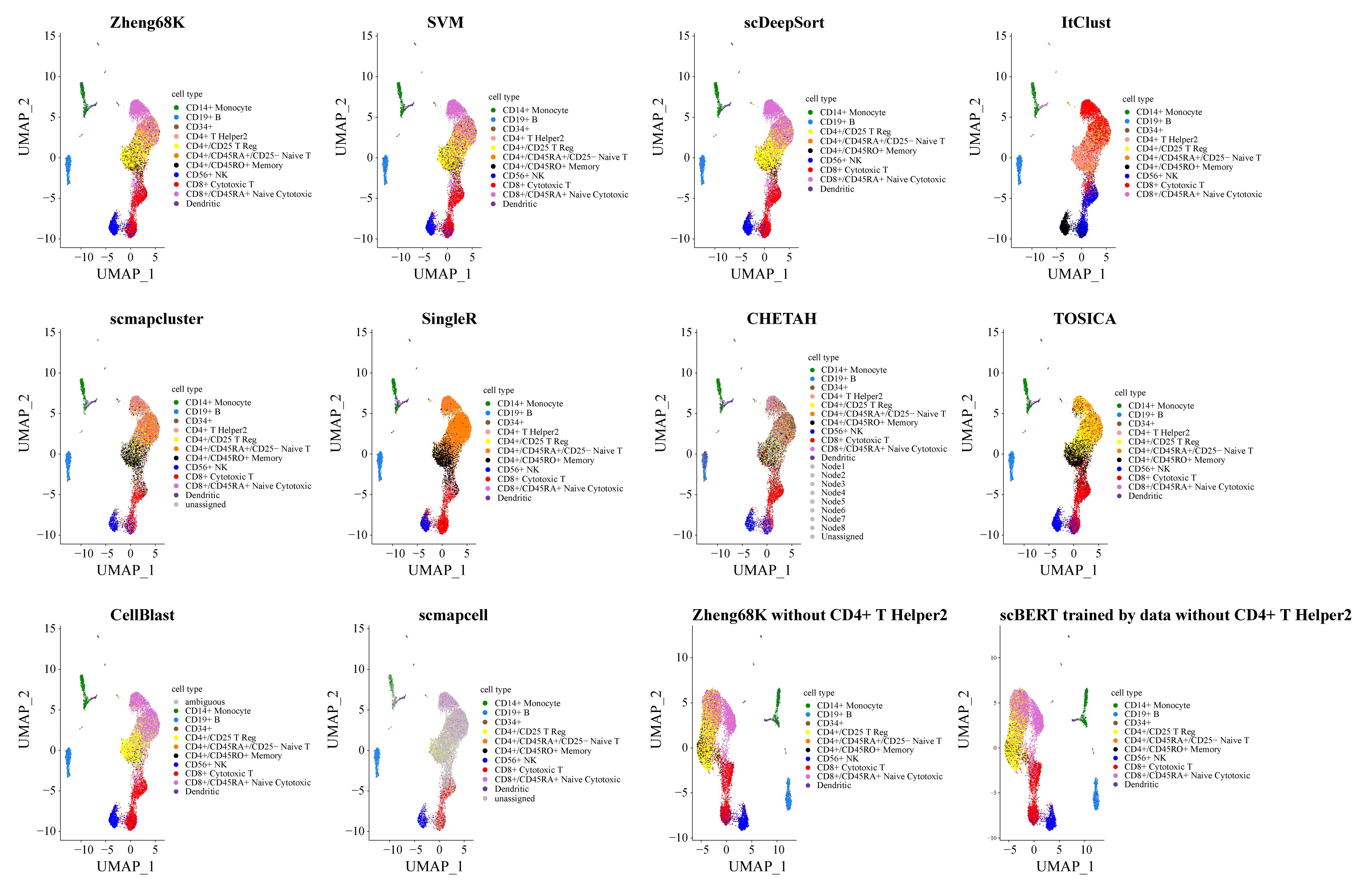

Supplement: Supplementary_Figure_8_bbae392 [file supplementary_figure_8_bbae392.jpeg]

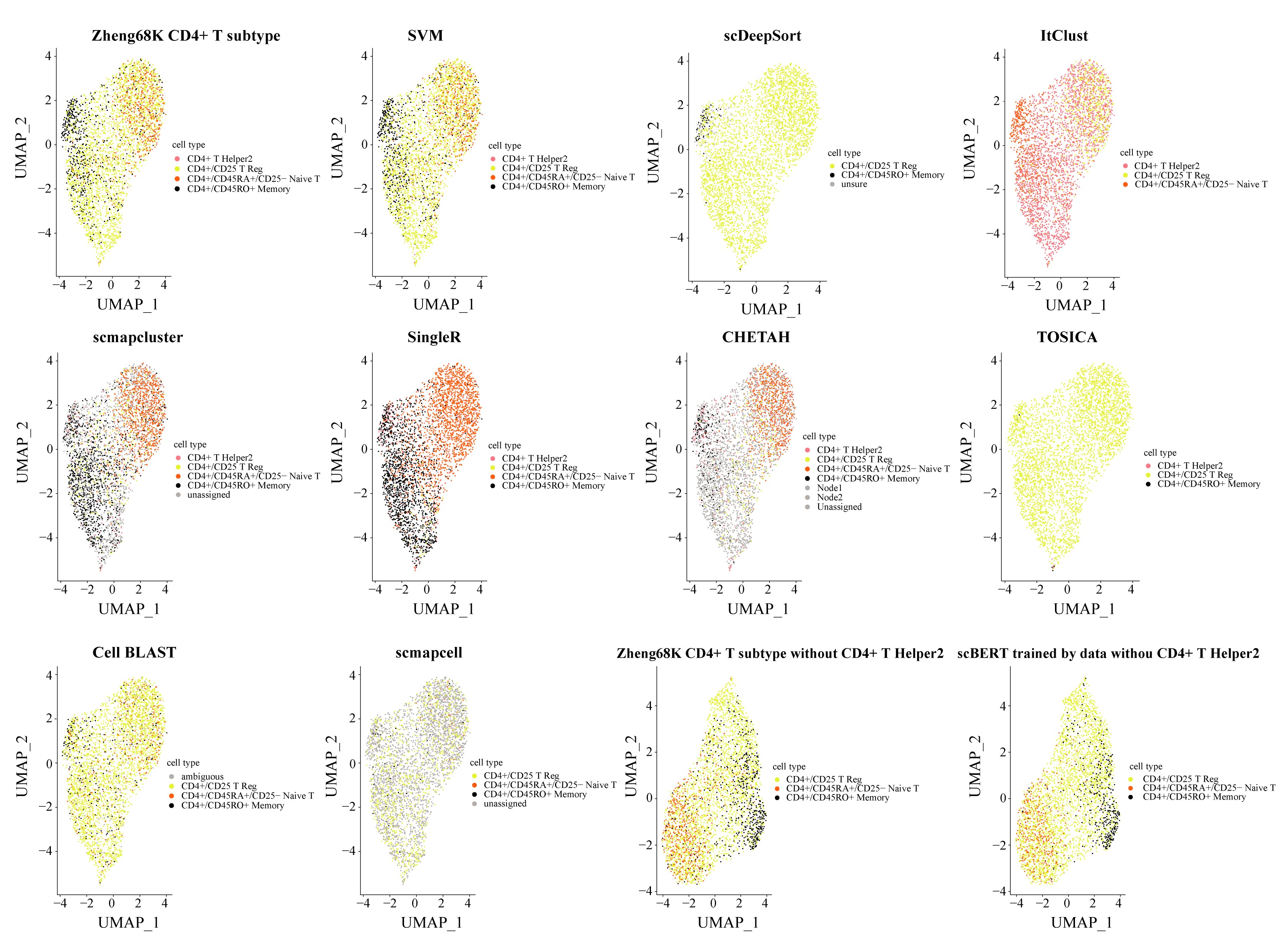

Supplement: Supplementary_Figure_9_bbae392 [file supplementary_figure_9_bbae392.jpeg]

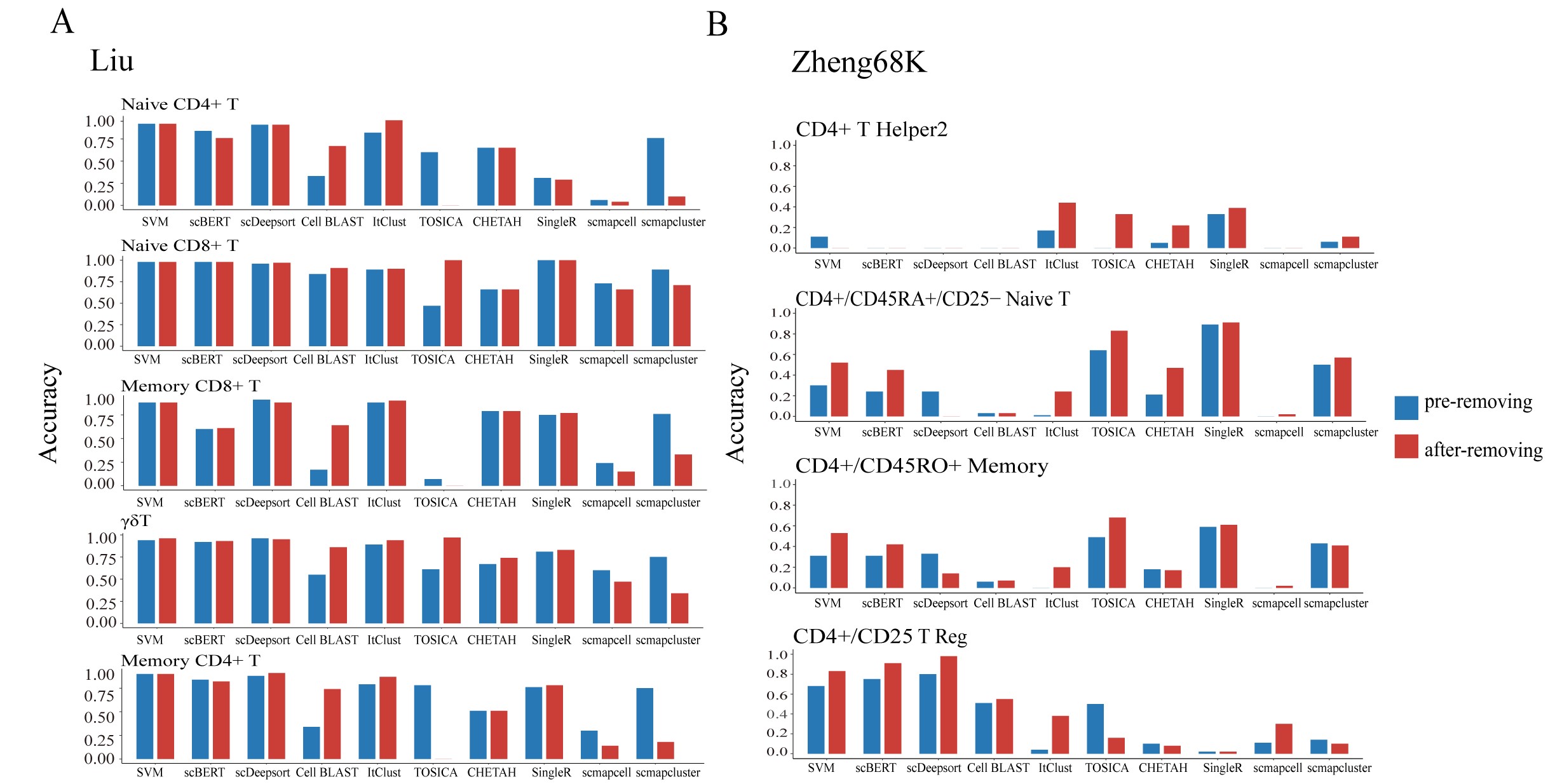

Supplement: Supplementary_Figure_10_bbae392 [file supplementary_figure_10_bbae392.jpeg]

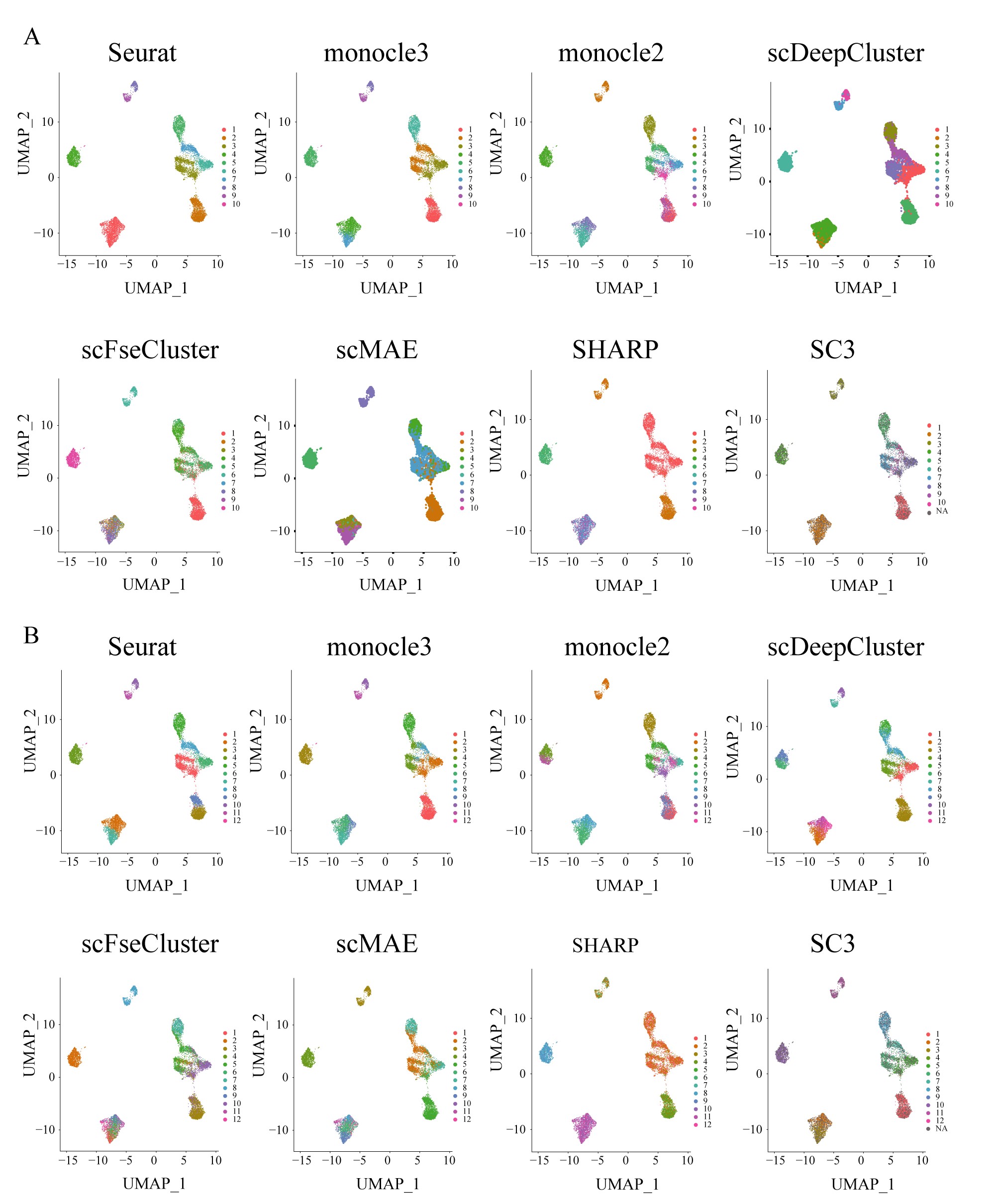

Supplement: Supplementary_Figure_11_bbae392 [file supplementary_figure_11_bbae392.jpeg]

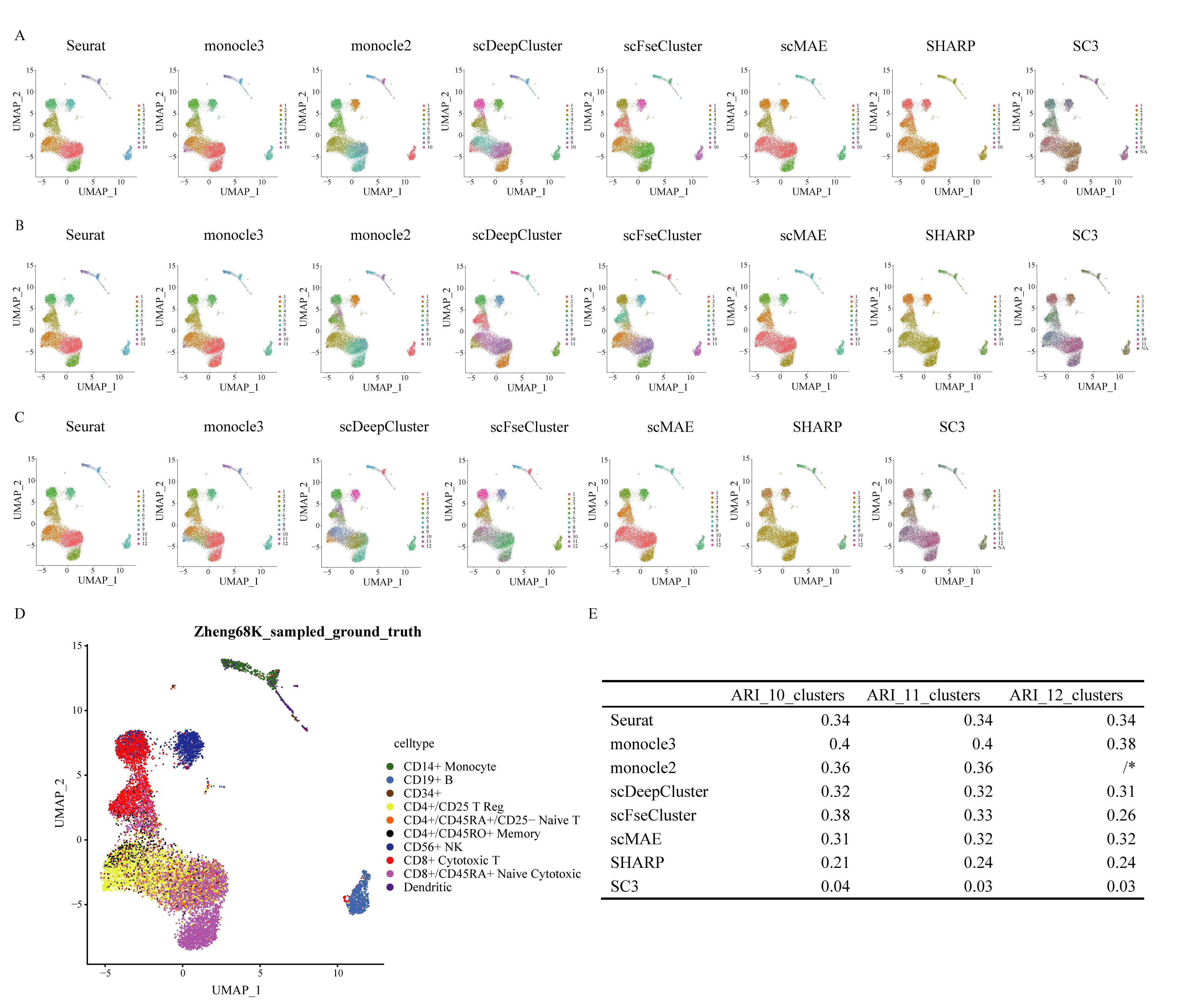

Supplement: Supplementary_Figure_12_bbae392 [file supplementary_figure_12_bbae392.jpeg]

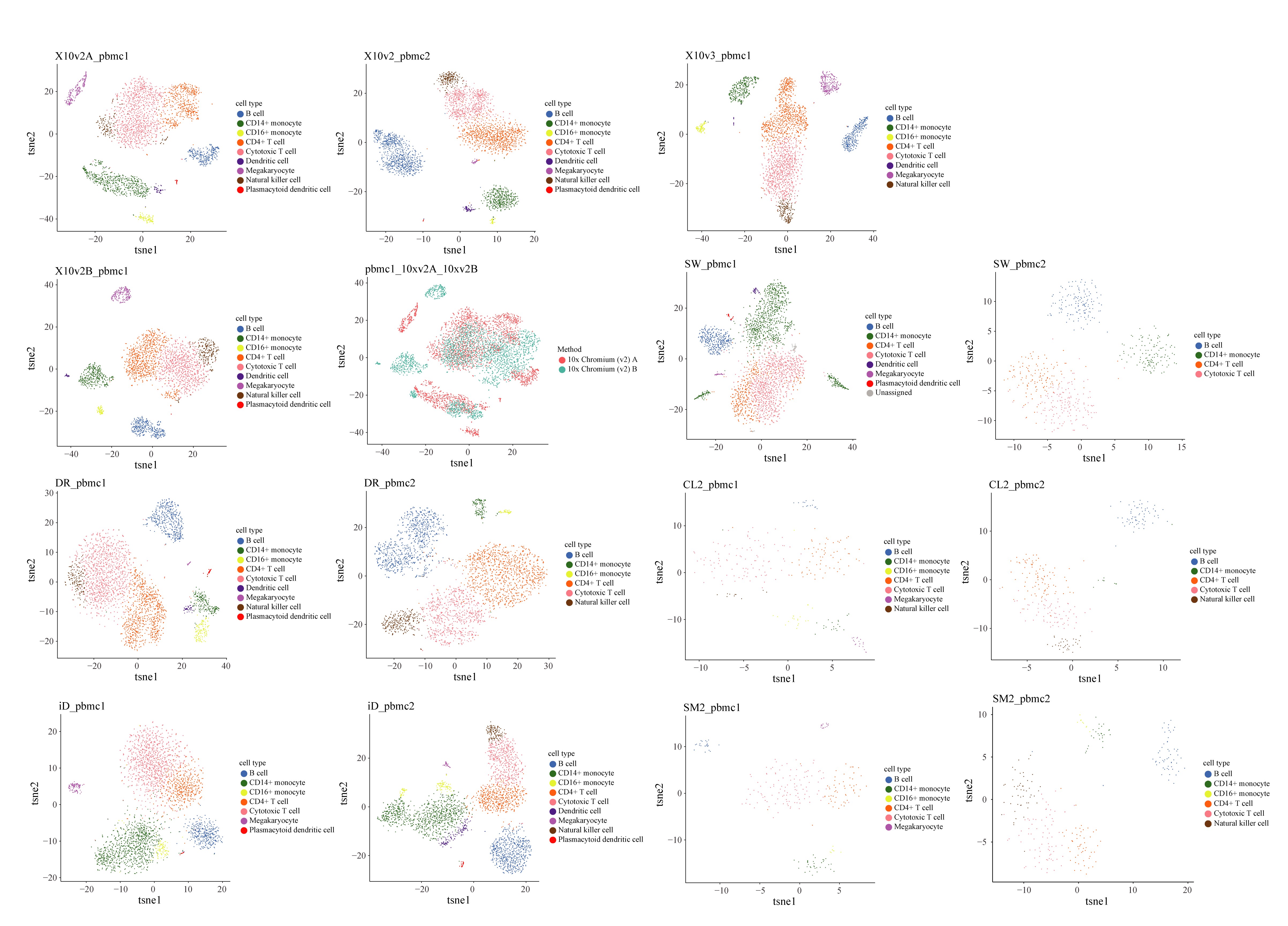

Supplement: Supplementary_Figure_13_bbae392 [file supplementary_figure_13_bbae392.jpeg]

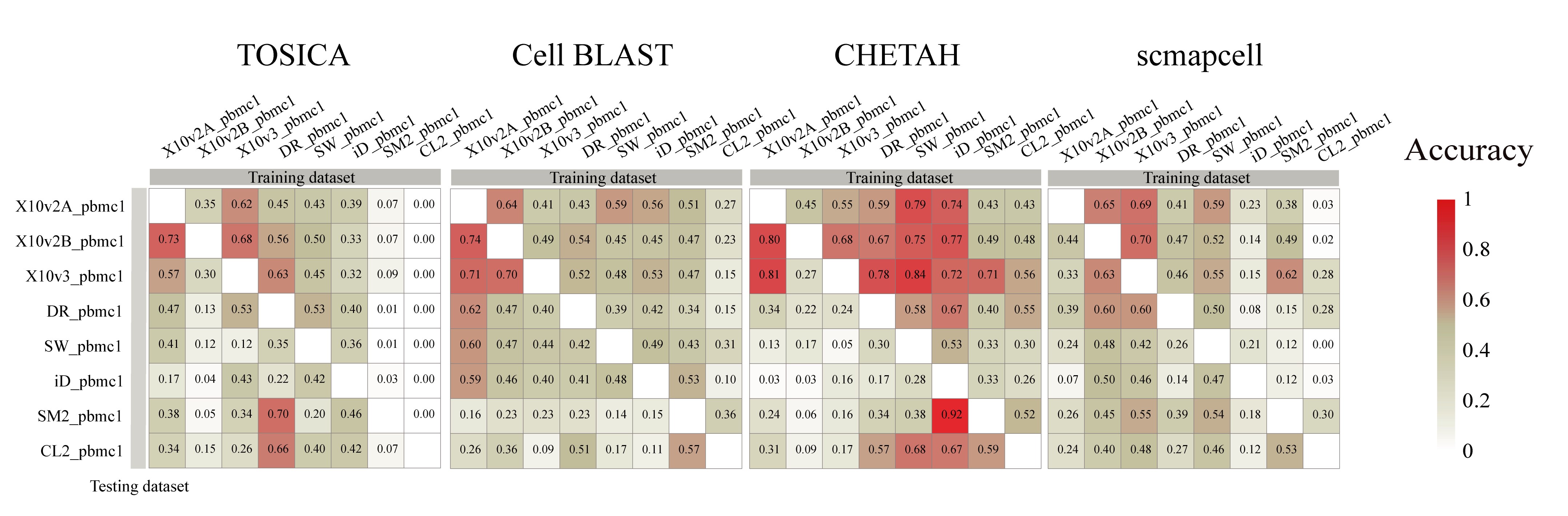

Supplement: Supplementary_Figure_14_bbae392 [file supplementary_figure_14_bbae392.jpeg]
