# Supplementary material for: A comparison of scRNA-seq annotation methods based on experimentally labeled immune cell subtype dataset
Source: Brief Bioinform. 2024 Aug 9;25(5):bbae392. doi: 10.1093/bib/bbae392 (PMC11312369; doi:10.1093/bib/bbae392)
Supplement: Supplementary_Table_bbae392 [file supplementary_table_bbae392.docx]

**Supplementary Table 1.** The correspondence between cell types and cell-type-specific sequences

| Tag name | Cell type | Sample Tag sequence |
| --- | --- | --- |
| Tag 1 | Naive CD4+ T | ATTCAAGGGCAGCCGCGTCACGAT  TGGATACGACTGTTGGACCGG |
| Tag 2 | Memory CD4+ T | TGGATGGGATAAGTGCGTGATGGA  CCGAAGGGACCTCGTGGCCGG |
| Tag 3 | Memory B | CGGCTCGTGCTGCGTCGTCTCAAG  TCCAGAAACTCCGTGTATCCT |
| Tag 4 | NK | ATTGGGAGGCTTTCGTACCGCTGC  CGCCACCAGGTGATACCCGCT |
| Tag 5 | Monocyte | CTCCCTGGTGTTCAATACCCGATGT  GGTGGGCAGAATGTGGCTGG |
| Tag 6 | Naive B | TTACCCGCAGGAAGACGTATACCC  CTCGTGCCAGGCGACCAATGC |
| Tag 7 | Naive CD8+ T | TGTCTACGTCGGACCGCAAGAAGT  GAGTCAGAGGCTGCACGCTGT |
| Tag 8 | Memory CD8+ T | CCCCACCAGGTTGCTTTGTCGG  ACGAGCCCGCACAGCGCTAGGAT |
| Tag 9 | Granulocyte | GTGATCCGCGCAGGCACACATAC  CGACTCAGATGGGTTGTCCAGG |
| Tag 10 | γδ T | GCAGCCGGCGTCGTACGAGGCAC  AGCGGAGACTAGATGAGGCCCC |

**Supplementary Table 2.** Information of immunomagnetic enrichment bead kits, immune cell surface proteins, theoretical purity of immune cells

| Cell type | Catalog and kit | Cell surface markers | Theoretical purity |
| --- | --- | --- | --- |
| Naive CD4+ T | 17555 EasySep™ Human Naive CD4+ T Cell Isolation Kit II | CD3+CD4+CD45RA+CD45RO- | 96.6 ± 1.5% |
| Memory CD4+ T | 19157 EasySep™ Human Memory CD4+ T Cell Enrichment Kit | CD4+CD45RA-CD45RO+ | 86 ­ 98% |
| Naive CD8+ T | 17968 EasySep™ Human Naive CD8+ T Cell Isolation Kit II | CD8+CD45RA+CCR7+ and CD45RO-CD57-CD56- | 93.7 ± 2.4% |
| Memory CD8+ T | 19159 EasySep™ Human Memory CD8+ T Cell Enrichment Kit | CD8+CD45RA-CD45RO+ | 72 - 92% |
| γδT | 19255 EasySep™ Human Gamma/Delta T Cell Isolation Kit | TCR gamma/delta+CD3+ | 90 - 97% |
| Naive B | 17864 EasySep™ Human Memory B Cell Isolation Kit | CD3-CD19+CD27- | 93 ± 5% |
| Memory B |  | CD19+CD27+ | 97 ± 2% |
| NK | 17955 EasySep™ Human NK Cell Isolation Kit | CD3-CD56+ | 85.0 ± 8.0% |
| Monocyte | 19359 EasySep™ Human Monocyte Isolation Kit | CD14+CD45+ | 89.7 ± 3.4% |
| Granulocyte | 19659 EasySep™ Direct Human Pan-Granulocyte Isolation Kit | Granulocytes (neutrophil [CD66b+CD16+], eosinophil [CD66b+CD16-] and basophil [CD66b-CD123+]) | 98.4 ± 1.5% |

**Supplementary Table 3.** Introduction of scRNA-seq datasets

| Dataset | Labels source | No. of cells | No. of genes | No. of cell populations | data type | Protocol |
| --- | --- | --- | --- | --- | --- | --- |
| Zhengsort | Bead-enriched | 20000 | 21952 | 10 | UMI count | 10 X Genomics |
| Zheng68K | Bead-enriched & spearman correlation | 65943 | 20387 | 11 | UMI count | 10 X Genomics |
| Liu | Bead-enriched | 9266 | 29007 | 10 | UMI count | Smart-seq2 |
| X10v2A_pbmc1 | Louvain community detection algorithm & AUC | 3222 | 33660 | 9 | UMI count | 10 X Genomics |
| X10v2B_pbmc1 | Louvain community detection algorithm & AUC | 3222 | 33660 | 9 | UMI count | 10 X Genomics |
| X10v2_pbmc2 | Louvain community detection algorithm & AUC | 3362 | 33660 | 9 | UMI count | 10 X Genomics |
| X10v3_pbmc1 | Louvain community detection algorithm & AUC | 3222 | 33660 | 8 | UMI count | 10 X Genomics |
| SW_pbmc1 | Louvain community detection algorithm & AUC | 3176 | 33660 | 7 | UMI count | Seqwell |
| SW_pbmc2 | Louvain community detection algorithm & AUC | 551 | 33660 | 4 | UMI count | Seqwell |
| DR_pbmc1 | Louvain community detection algorithm & AUC | 3222 | 33660 | 9 | UMI count | Drop-seq |
| DR_pbmc2 | Louvain community detection algorithm & AUC | 3362 | 33660 | 6 | UMI count | Drop-seq |
| iD_pbmc1 | Louvain community detection algorithm & AUC | 3222 | 33660 | 7 | UMI count | inDrops-seq |
| iD_pbmc2 | Louvain community detection algorithm & AUC | 3362 | 33660 | 9 | UMI count | inDrops-seq |
| CL2_pbmc1 | Louvain community detection algorithm & AUC | 253 | 33660 | 7 | UMI count | Celseq2 |
| CL2_pbmc2 | Louvain community detection algorithm & AUC | 273 | 33660 | 5 | UMI count | Celseq2 |
| SM2_pbmc1 | Louvain community detection algorithm & AUC | 253 | 33660 | 6 | read count | Smart-seq2 |
| SM2_pbmc2 | Louvain community detection algorithm & AUC | 273 | 33660 | 6 | read count | Smart-seq2 |

**Supplementary Table 4.** Datasets commonly used in the evaluation of annotation methods

| Dataset | Description | Labels source |
| --- | --- | --- |
| Zhengsort[1] | Peripheral blood cell | Bead-enriched PBMC subpopulations validated by FACS |
| Zheng68K[2] | Peripheral blood cell | Spearman correlation |
| Baron (Mouse)[3] | Mouse pancreas | an iterative hierarchical clustering method |
| Baron (Human)[3] | Human pancreas | an iterative hierarchical clustering method |
| Muraro[4] | Human pancreas | Clustering by StemID |
| Segerstolpe[5] | Human pancreas | tSNE |
| Xin[6] | Human pancreas | unsupervised hierarchical clustering |
| CellBench 10X[7] | Mixture of five human lung cancer cell lines sequenced by 10X Chromium | Labels are identified based known genetic variation |
| CellBench CEL-Seq2[7] | Mixture of five human lung cancer cell lines sequenced by CEL-seq2 | Labels are identified based known genetic variation |
| Tm[8] | Whole Mus musculus | annotation based on the nearest-neighbor graph-based clustering & Differential analysis & known markers |
| AMB[9] | Primary mouse visual cortex | iterative clustering & DE analysis |

**Supplementary Table 5.** The composition and number of cell types in different datasets

| Dataset | Cell type | | Number |
| --- | --- | --- | --- |
| Liu | γδ T | | 1395 |
|  | Granulocyte | | 1972 |
|  | Memory B | | 256 |
|  | Memory CD4+ T | | 938 |
|  | Memory CD8+ T | | 554 |
|  | Monocyte | | 1084 |
|  | Naive B | | 287 |
|  | Naive CD4+ T | | 594 |
|  | Naive CD8+ T | | 1160 |
|  | NK | | 1026 |
| Zheng68K | CD14+ Monocyte | | 2376 |
|  | CD19+ B | | 3694 |
|  | CD34+ | | 177 |
|  | CD4+ T Helper2 | | 18 |
|  | CD4+/CD25 T Reg | | 13874 |
|  | CD4+/CD45RA+/CD25- Naive T | | 2769 |
|  | CD4+/CD45RO+ Memory | | 3071 |
|  | CD56+ NK | | 5666 |
|  | CD8+ Cytotoxic T | | 11157 |
|  | CD8+/CD45RA+ Naive Cytotoxic | | 21445 |
|  | Dendritic | | 1696 |
| ZhengSort | CD14+ Monocyte | | 2000 |
|  | CD19+ B | | 2000 |
|  | CD34+ | | 2000 |
|  | CD4+ T Helper2 | | 2000 |
|  | CD4+/CD25 T Reg | | 2000 |
|  | CD4+/CD45RA+/CD25- Naive T | | 2000 |
|  | CD4+/CD45RO+ Memory | | 2000 |
|  | CD56+ NK | | 2000 |
|  | CD8+ Cytotoxic T | | 2000 |
|  | CD8+/CD45RA+ Naive Cytotoxic | | 2000 |
| X10v2B_pbmc1 | B cell | | 388 |
|  | CD14+ monocyte | | 379 |
|  | CD16+ monocyte | | 73 |
|  | CD4+ T cell | | 908 |
|  | Cytotoxic T cell | | 954 |
|  | Dendritic cell | | 33 |
|  | Megakaryocyte | | 212 |
|  | Natural killer cell | | 263 |
|  | Plasmacytoid dendritic cell | | 12 |
| X10v2A_pbmc1 | B cell | | 288 |
|  | CD14+ monocyte | | 640 |
|  | CD16+ monocyte | | 102 |
|  | CD4+ T cell | | 550 |
|  | Cytotoxic T cell | | 1174 |
|  | Dendritic cell | | 55 |
|  | Megakaryocyte | | 221 |
|  | Natural killer cell | | 166 |
|  | Plasmacytoid dendritic cell | | 26 |
| X10v2_pbmc2 | B cell | | 862 |
|  | CD14+ monocyte | | 436 |
|  | CD16+ monocyte | | 50 |
|  | CD4+ T cell | | 963 |
|  | Cytotoxic T cell | | 694 |
|  | Dendritic cell | | 76 |
|  | Megakaryocyte | | 32 |
|  | Natural killer cell | | 219 |
|  | Plasmacytoid dendritic cell | | 30 |
| X10v3_pbmc1 | B cell | | 346 |
|  | CD14+ monocyte | | 354 |
|  | CD16+ monocyte | | 98 |
|  | CD4+ T cell | | 960 |
|  | Cytotoxic T cell | | 962 |
|  | Dendritic cell | | 38 |
|  | Megakaryocyte | | 270 |
|  | Natural killer cell | | 194 |
| SW_pbmc1 | B cell | | 387 |
|  | CD14+ monocyte | | 1132 |
|  | CD4+ T cell | | 435 |
|  | Cytotoxic T cell | | 1121 |
|  | Dendritic cell | | 37 |
|  | Megakaryocyte | | 38 |
|  | Plasmacytoid dendritic cell | | 26 |
| SW_pbmc2 | B cell | | 140 |
|  | CD14+ monocyte | | 123 |
|  | CD4+ T cell | | 131 |
|  | Cytotoxic T cell | | 157 |
| DR_pbmc1 | B cell | | 477 |
|  | CD14+ monocyte | | 232 |
|  | CD16+ monocyte | | 149 |
|  | CD4+ T cell | | 889 |
|  | Cytotoxic T cell | | 1189 |
|  | Dendritic cell | | 46 |
|  | Megakaryocyte | | 27 |
|  | Natural killer cell | | 185 |
|  | Megakaryocyte | | 28 |
| DR_pbmc2 | B cell | | 928 |
|  | CD14+ monocyte | | 125 |
|  | CD16+ monocyte | | 49 |
|  | CD4+ T cell | | 1172 |
|  | Cytotoxic T cell | | 789 |
|  | Natural killer cell | | 299 |
| iD_pbmc1 | B cell | | 374 |
|  | CD14+ monocyte | | 1006 |
|  | CD16+ monocyte | | 108 |
|  | CD4+ T cell | | 512 |
|  | Cytotoxic T cell | | 1108 |
|  | Megakaryocyte | | 104 |
|  | Plasmacytoid dendritic cell | | 10 |
| iD_pbmc2 | B cell | | 671 |
|  | CD14+ monocyte | | 1032 |
|  | CD16+ monocyte | | 136 |
|  | CD4+ T cell | | 599 |
|  | Cytotoxic T cell | | 556 |
|  | Dendritic cell | | 148 |
|  | Megakaryocyte | | 41 |
|  | Natural killer cell | | 147 |
|  | Plasmacytoid dendritic cell | | 32 |
| CL2_pbmc1 | B cell | | 14 |
|  | CD14+ monocyte | | 20 |
|  | CD16+ monocyte | | 21 |
|  | CD4+ T cell | | 67 |
|  | Cytotoxic T cell | | 103 |
|  | Megakaryocyte | | 18 |
|  | Natural killer cell | | 10 |
| CL2_pbmc2 | B cell | | 66 |
|  | CD14+ monocyte | | 11 |
|  | CD4+ T cell | | 93 |
|  | Cytotoxic T cell | | 71 |
|  | Natural killer cell | | 32 |
| SM2_pbmc1 | B cell | | 22 |
|  | CD14+ monocyte | | 34 |
|  | CD16+ monocyte | | 8 |
|  | CD4+ T cell | | 58 |
|  | Cytotoxic T cell | | 117 |
|  | Megakaryocyte | | 14 |
| SM2_pbmc2 | B cell | | 57 |
|  | | CD14+ monocyte | 26 |
|  | | CD16+ monocyte | 10 |
|  | | CD4+ T cell | 54 |
|  | | Cytotoxic T cell | 76 |
|  | | Natural killer cell | 50 |

**Reference**

1. Abdelaal T, Michielsen L, Cats D et al. A comparison of automatic cell identification methods for single-cell RNA sequencing data, Genome Biol 2019;20:194.

2. Zheng GX, Terry JM, Belgrader P et al. Massively parallel digital transcriptional profiling of single cells, Nat Commun 2017;8:14049.

3. Baron M, Veres A, Wolock SL et al. A Single-Cell Transcriptomic Map of the Human and Mouse Pancreas Reveals Inter- and Intra-cell Population Structure, Cell Syst 2016;3:346-360.e344.

4. Muraro MJ, Dharmadhikari G, Grün D et al. A Single-Cell Transcriptome Atlas of the Human Pancreas, Cell Syst 2016;3:385-394.e383.

5. Segerstolpe Å, Palasantza A, Eliasson P et al. Single-Cell Transcriptome Profiling of Human Pancreatic Islets in Health and Type 2 Diabetes, Cell Metab 2016;24:593-607.

6. Xin Y, Kim J, Okamoto H et al. RNA Sequencing of Single Human Islet Cells Reveals Type 2 Diabetes Genes, Cell Metab 2016;24:608-615.

7. Tian L, Dong X, Freytag S et al. Benchmarking single cell RNA-sequencing analysis pipelines using mixture control experiments, Nat Methods 2019;16:479-487.

8. Single-cell transcriptomics of 20 mouse organs creates a Tabula Muris, Nature 2018;562:367-372.

9. Tasic B, Yao Z, Graybuck LT et al. Shared and distinct transcriptomic cell types across neocortical areas, Nature 2018;563:72-78.
